# Evaluation of Creep Behavior of Soft Soils by Utilizing Multisensor Data Combined with Machine Learning

**DOI:** 10.3390/s22082888

**Published:** 2022-04-09

**Authors:** Meho Saša Kovačević, Mario Bačić, Lovorka Librić, Kenneth Gavin

**Affiliations:** 1 Faculty of Civil Engineering, University of Zagreb, 10000 Zagreb, Croatia; msk@grad.hr (M.S.K.); llibric@grad.hr (L.L.); 2 Faculty of Civil Engineering and Geosciences, TU Delft, 2628 CN Delft, The Netherlands; k.g.gavin@tudelft.nl

**Keywords:** soft soil creep, Burger’s model, neural network, particle swarm optimization, remote sensing, nondestructive testing

## Abstract

To identify the unknown values of the parameters of Burger’s constitutive law, commonly used for the evaluation of the creep behavior of the soft soils, this paper demonstrates a procedure relying on the data obtained from multiple sensors, where each sensor is used to its best advantage. The geophysical, geotechnical, and unmanned aerial vehicle data are used for the development of a numerical model whose results feed into the custom-architecture neural network, which then provides information about on the complex relationships between the creep characteristics and soil displacements. By utilizing InSAR and GPS monitoring data, particle swarm algorithm identifies the most probable set of Burger’s creep parameters, eventually providing a reliable estimation of the long-term behavior of soft soils. The validation of methodology is conducted for the Oostmolendijk embankment in the Netherlands, constructed on the soft clay and peat layers. The validation results show that the application of the proposed methodology, which relies on multisensor data, can overcome the high cost and long duration issues of laboratory tests for the determination of the creep parameters and can provide reliable estimates of the long-term behavior of geotechnical structures constructed on soft soils.

## 1. Introduction

**Figure 1:**
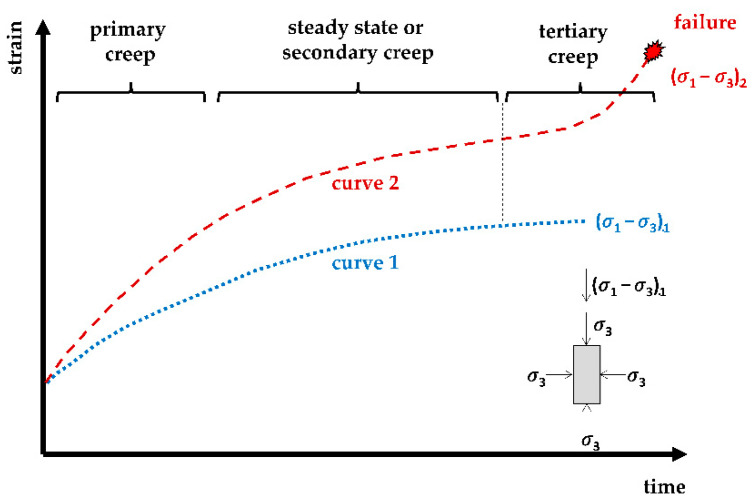
Different phases of creep in various stress regime, redrawn from [7].

Shortly after Terzaghi [1] developed his well-known soil consolidation theory, Buisman [2] proposed a creep law for soft soils based on observations that their settlements could not be fully explained by classical consolidation theory. These ‘soft soils’ usually include near-normally consolidated clays, clayey silts, and peats, which are characterized by their high degree of compressibility. Since then, many researchers [3,4,5] have dealt with the creep phenomenon. However, as Kaczmarek and Dobak [6] note, despite many attempts to clarify this phenomenon, there is still no explanation of the reasons for soil creep movement. This leads to the general definition of soil creep as increase of time-dependent strains at the constant stress levels, meaning that soft soils continue to settle even after the primary consolidation is deemed to be finished. Three main creep phases can be identified, including the primary, secondary, and tertiary creep, Figure 1. While the major stress can be small enough so that the creep change of the soil structure is decelerated (curve 1), the creep stress could also lead to excessive deformation and eventually to the soil failure (curve 2).

Disagreements in the scientific community still arise when trying to explain the genesis and the time occurrence of the creep phenomenon. Two hypotheses are discussed: (1) where the creep phenomenon occurs from the beginning, along with the soil primary consolidation and (2) where the creep phenomenon starts after the end of primary consolidation. Kaczmarek and Dobak [6] note that several studies have confirmed the first hypothesis in which the soil void ratio is affected by the thickness of the soil layer and drainage conditions; however, due to the complexity in settlement calculation, the second hypothesis is still mostly used in geotechnical practice as an initial approximation of the creep behavior.

The issues of soft soil creep usually come to the fore after the excessive settlements or failures of geotechnical structures constructed on them. Back in the 1960s, Bjerrum [3] noted that time dependency may be a significant contributor to the performance of geotechnical structures. According to Veremeer [7], substantial creep settlements in later years is pronounced for road or river embankments on soft soils, whose construction yields large primary compression, assuming that the secondary compression is a small percentage of the primary compression. This was confirmed by several studies [8,9,10] on well-instrumented embankments constructed on soft soils, which experienced large deformations long after they have been constructed. These long-term deformations are one of the main reasons for millions of dollars spent annually for the maintenance of the road embankments or other geotechnical structures constructed on soft soils [11].

The development of the computers and numerical techniques during the 1990s significantly eased practical implementations of constitutive models that incorporate creep effects. This provided both researchers and practitioners with the possibility to predict the behavior of the time-dependent and long-term behavior of soft soil. The constitutive models, which include creep, overcame the incapacities of classical elastoplastic models to capture soil creep behavior. Some of these viscoplastic (EVP)-based models are the soft-soil creep model [12], creep-SCLAY1S [13], or modified Mesri creep model [14]. However, due to their unconvincing benefits and the complexity, cost, and time associated with the analyses [11], practical engineers appear reluctant to incorporate these models in practical field cases. In addition, these models require knowledge of the creep parameters, which are usually obtained by the high cost and long duration of laboratory tests. Moreover, very few of these models [15,16,17,18] have been tested against long-term field monitoring data. Verifications of the creep models on actual field cases are very important, since unlike primary compression, secondary compression or creep is often underestimated [19]. The purpose of this study is to demonstrate how can soil creep behavior be estimated by utilizing the continuous on-site displacement measurements, thus overcoming the issues of laboratory creep tests and nonreliability of empirical models. For this, a neural network (NN) tool will be used. Due to their superiority over the traditionally used statistical and experimental methods, NNs have been extensively used in field of geotechnical engineering [20,21,22,23,24,25,26]. Among their numerous advantages, a remarkable information processing capability pertinent to nonlinearity is of a highest benefit. As Reale et al. [27] note, by sharing information between interconnected artificial neural network elements, complex relationships that are intuitively difficult to describe can be established. This is similar to the behavior of the human brain and the nervous system, thus the name ‘neural network’.

Many authors have recognized the benefits of using the NNs to determine creep related parameters of both soft rocks [26,28,29] and soft soils [30,31,32,33], thus overcoming the mentioned issues of laboratory tests. For example, Zhang et al. [30] proposed a hybrid surrogate intelligent model for predicting the soft soil creep index (C_α_), motivated by the fact that current empirical models for C_α_ determination are not sufficiently reliable. Within this study, the authors collected datasets of four input soil parameters determined by the simple laboratory tests and defined C_α_ as the NN output parameter. The developed models demonstrably outperformed empirical methods, featuring as they do lower levels of prediction error. Liu et al. [31] demonstrated that the creep models can avoid artificial assumption of complex constitutive equation and can reflect nonlinear creep properties of soft soil objectively. Further, Chen et al. [32] developed an NN-based model, by utilizing the creep data of a laboratory direct shear experiment. The modeling method was validated on the creep experimental data of soft clay of Shanghai, where the study clearly demonstrated that the rheological model can effectively describe the nonlinear creep of soft clay. To assess the soil creep susceptible areas, Lee et al. [33] used several machine learning classification methods, namely the k-nearest neighbor (k-NN), naive bayes (NB), random forest (RF), and support vector machine (SVM) models, where results from almost 5000 field surveys were utilized for the development of the classification models. The benefit of the NNs is also evident in the studies for predicting the compressibility parameters of the soils, which govern the soft soil behavior before it reaches the creep phase. For example, Kurnaz et al. [34] suggested the prediction of compressibility parameters from basic soil properties, concluding that the proposed NN model is successful for the prediction of the compression index, with less accuracy in prediction of recompression index values.

This study predicts the creep parameters for a constitutive model based on a combination of multisensor data that feed into the numerical model. The numerical simulation input–output datasets were then used for the development of the NN, which provides information about the complex relationships between the creep characteristics and soil settlements and where the continuous on-site displacement measurements enabled identification of the most probable set of creep parameters. The overall methodology was validated in a case study of an Oostmolendijk embankment in the Netherlands, as a well-known example of a continuously settling geotechnical structure, where the validation procedure included the implementation of a developed NN for the prediction of the embankment’s displacements based on the long-term InSAR and GPS monitoring data.

## 2. Methods and Methodology of Soil Creep Prediction Based on Multisensor Data

The focal point of the methodology is in estimation of the soft soil creep parameters by using machine learning supported with the multisensor data, where each sensor can be used to its best advantage in the different phases of methodology.

### 2.1. Constitutive Law Used for Numerical Modeling

**Figure 2:**
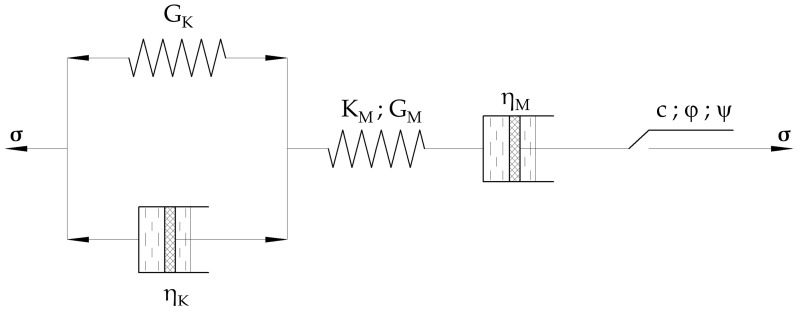
A classic Burger’s creep viscoplastic model, redrawn from [35].

The methodology is based on the numerical modeling and application of the classic Burger’s creep viscoplastic constitutive model, Figure 2, represented by a series of spring, dashpot, and plastic sliders that are connected in parallel and/or in series. More precisely, the constitutive model consists of viscoelastic part, where Kelvin’s unit (characterized by its shear modulus G_K_ and viscosity η_K_) and Maxwell’s unit (characterized by its shear elastic shear modulus G_M_, viscosity η_M_, and bulk modulus K_M_) act in series. In addition, a plastic strain–rate part, which utilizes the Mohr–Coulomb failure criteria and is characterized by the cohesion (c), friction angle (ϕ), and dilation (ψ), is connected in series with viscoelastic part of model.

To reliably describe the different elements of this constitutive model, their proper evaluation is of the highest importance. While the strength and stiffness parameters can be easily obtained by common laboratory tests or by utilizing established correlations with in situ investigation results, determining Kelvin’s and Maxwell’s viscosity parameters (η_K_ and η_M_) is a challenging task. Therefore, these two parameters represent this study’s first two constitutive unknowns. Further, several stiffness values are required by the Burger’s model, and these include Maxwell’s bulk and shear modulus (K_M_ and G_M_) and Kelvin’s shear modulus (G_K_). By utilizing well-known equations:

(1) $$G_{M,0}=\rho \cdot {\;v}_{s}^{2}$$

(2) $$K_{M,0}=\frac{2\cdot G_{M,0}\cdot \left(1+\nu \right)}{3\cdot \left(1-2\nu \right)}$$

where G_M,0_ and K_M,0_ are Maxwell’s small-strain shear and bulk modulus (in Pa), respectively, ρ (kg/m^3^) is soil density, determined in this study from continuous-by-depth CPT procedure developed by Kovačević et al. [36], v_s_ (m/s) is a soil shear velocity obtained in this study from geophysical MASW investigations, and ν (-) is Poisson’s ratio. Since creep mechanism involves large-strain moduli, which are lower than the small-strain values, these could be obtained by introducing the so-called reduction radio (r_d_), which in fact represents the percentage of small-strain modulus and can be defined as:

(3) $$G_{M}=r_{d}\;\cdot {\;G}_{M,0}$$

(4) $$K_{M}=r_{d}\;\cdot {\;K}_{M,0}$$

where G_M_ and K_M_ are Maxwell’s large-strain shear and bulk modulus (in Pa). Therefore, a reduction ratio represents the third unknown of the creep estimation methodology, which correlates with the required large-strain Burger’s moduli with the known values of small-strain moduli. It should be noted that Kelvin’s shear modulus (G_K_) was assumed as zero within this methodology since it acts in series with Maxwell’s shear modulus (G_M_), so that the shear modulus dependence of the system could be assigned only to Maxwell’s shear modulus.

To sum up, as an alternative to the high cost and long duration of laboratory tests, which include creep testing, this study offers a solution for identifying constitutive model creep unknowns of stiffness reduction ratio (r_d_), Kelvin viscosity (η_K_), and Maxwell viscosity (η_M_), by utilizing the neural network along with the application of the particle swarm optimization (PSO) algorithm.

### 2.2. Methodology Phases

**Figure 3:**
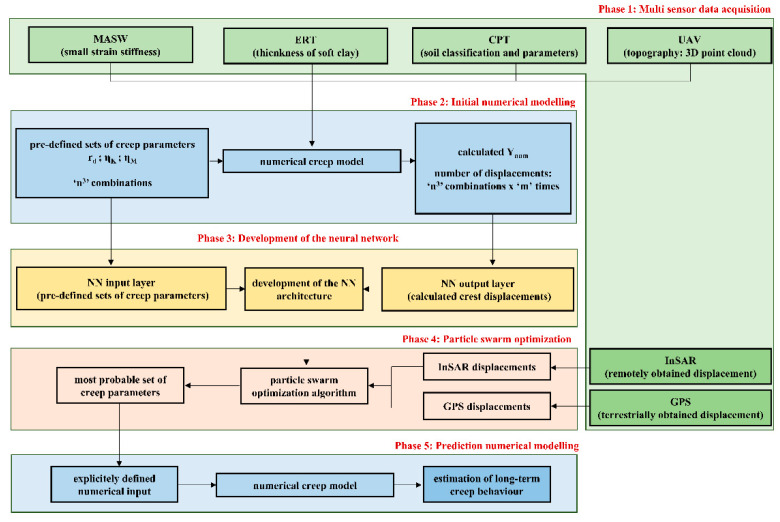
A framework for estimation of long-term creep behavior of soft soils.

The overall methodology for the identification of the most probable set of identified unknowns, included several phases, as indicated in Figure 3.

The first phase included acquisition of multisensor data necessary for the development of a reliable numerical model, including its geometrical and physical–mechanical characteristics. Here, unmanned aerial vehicle (UAV) scanning of the area provided the 3D point cloud of the terrain by means of the photogrammetry technique. By doing a relatively simple scanning of the area, a lot of useful information could be obtained, including the extraction of preferable cross-section of the terrain used for numerical model. In addition to having the UAV topography information, it was of upmost importance to obtain information on subsoil condition. This study utilized the cone penetration test (CPT) for the classification of soil, as well for the determination of the soil’s physical and mechanical characteristics relevant for the numerical analysis. Since CPT provides discrete information (in one point), additional geophysical investigations of multichannel analysis of surface waves (MASW) and electrical tomography (ERT) are utilized.

Once the numerical model was developed on the basis of acquired data, the second methodology phase included the generation of a database with a large number (n^3^, with ‘n’ being the selected number of values for each parameter) of predefined input creep parameters (r_d_, η_K_, and η_M_). The range of these parameters should be selected in such way so that upper and lower boundary of each parameter range can be considered sufficient to estimate the most probable parameter value in the subsequent phases. These sets of parameters were applied to the numerical simulations, eventually resulting in n^3^ output sets, representing soil displacement values over a certain predefined period of time. For example, if five (5) possible values of three (3) unknowns were considered, this resulted in 125 (i.e., 5^3^) numerical simulations and 125 output displacements in each observed period. It should be noted that the numerical simulations in this case were time-dependent simulations, where a selection of time-step was necessary to ensure the stability of the time-dependent numerical solution.

After obtaining the input–output datasets from the conducted numerical simulations, the development of a NetCREEP neural network followed (‘net’ standing for ‘network’), using the back propagation learning algorithm (third phase). After a certain number of training iterations, the post-trained network was expected to approximate the aforementioned numerical simulations, that is to establish a complex correlation between the constitutive model creep unknowns and the time-dependent displacements. The iterative process was necessary to develop an optimal architecture of the NN. Usually, the number of necessary input–output datasets for this procedure depends both on the complexity of problem and on the complexity of the chosen algorithm.

The in situ long-term monitoring displacements, obtained in this study by the means of InSAR and GPS measurements, were then implemented as an input in the developed NetCREEP to determine the output as the ‘most probable set of creep (r_d_, η_K_, and η_M_) parameters’. By utilizing the particle swarm optimization (PSO) algorithm in the fourth phase, the best fitting curve, described by one set of parameters, was identified. This ‘most probable’ set of creep parameters was implemented in a developed numerical model to evaluate how the numerical model correlated with the obtained in situ measurement data. Finally, after obtaining a reliable estimation of the constitutive model creep parameters, the fifth phase of the methodology included a prediction of the long-term creep behavior of the time-dependent numerical model for any user-defined period.

### 2.3. Development of NetCREEP Neural Network and PSO Optimization

To develop the relationship between the creep parameters and soft soil displacements, a so-called multilayer layer perceptron (MLP) custom-made architecture was used within this study. In this type of NN, the input layer, a hidden layer (or hidden layers) and an output layer, each consisting of several neurons, were selected. The reader is directed to relevant reference [37] on the techniques for the selection of appropriate number of hidden layers and neurons, since this significantly influences NN performance. The assignment and adaptation of the weighting to each neuron interconnection provided development of the NN prediction capabilities. For this procedure to be successful, as proposed by Hammerstrom [38], 70% of total ‘creep parameter input’ and ‘displacement output’ data were used for training process in order is to find optimum neural weightings; 15% of total data was used for the following validation phase, which included simulation of output data with input data, and remaining 15% of total data was used for testing phase, conducted to provide an unbiased evaluation of a final model fit on the training dataset. The developed NetCREEP neural network provided information about the relationships between soil creep parameters and soil time-dependent displacements.

**Figure 4:**
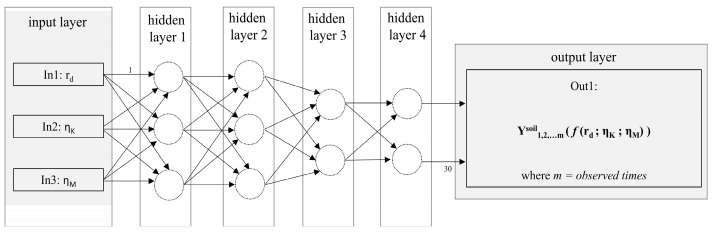
A scheme of a NetCREEP neural network.

While the NN input set consisted of ‘n’ values of selected creep parameters [r_d1_, r_d2_, …, r_dn_]; [η_K1_, η_K2_, …, η_Kn_]; [η_M1_, η_M2_, …, η_Mn_], the output was determined through the n^3^ displacements resulting from n^3^ numerical simulation. Therefore, the displacement was defined by the form of [y_t1_, y_t2_, …, y_tm_]_1_; [y_t1_, y_t2_, …, y_tm_]_2_; …; [y_t1_, y_t2_, …, y_tm_]_n_^3^ with ‘y’ being the displacement, ‘t’ being the observed time, and ‘m’ being the largest observed time. The developed NetCREEP consisted of four (4) hidden layers. The first and second hidden layers consisted of three (3) nodes, while the third and fourth consisted of two (2) nodes. In total, 30 distinct weightings were used, where a sigmoid activation function was utilized for hidden neurons, and a linear activation function was utilized for output, see Figure 4. The final number of hidden layers and the number of nodes in each hidden layer, was determined through a ‘trial and error’ method. Several ‘trial and error’ rule-of-thumb methods are described in the relevant literature [37]. In this study, by using the ‘trial and error’ method, 28 different NN architectures were analyzed, having a different number of hidden layers and associated number of nodes in each hidden layer. The selected architecture, from Figure 4, yielded largest values of R^2^, meaning that it established the highest strength of the ‘creep parameter-time-dependent displacement’ relationship.

The developed NetCREEP was further used for the identification of the most probable set of creep parameters through utilization of particle swarm optimization (PSO). The application of PSO, as a heuristic search method inspired by the collaborative behavior of biological populations, was first published by Kennedy and Eberhart [39], and it relies on particles randomly moving from points to another set of points in a single iteration, where the search procedures are improved using a combination of deterministic and probabilistic rules. As such it has been used for inverse parameter identification in some previous geotechnical studies [26,40,41]. In these studies, it was shown that particle swarms wee a fast and efficient tool for finding unknown parameter sets to represent the measured reference data. For this study, algorithm code was written within the Matlab software [42], where the PSO was applied to identify the most probable set of creep parameter values (r_d_, η_K_, and η_M_), which matched the measured InSAR and GPS displacements. As described by Jahed Armaghani et al. [43], the overall swarm size has large influence on the PSO performance, where this study utilized the size of 60 particles.

## 3. Investigation Methods and Multisensor Data

The multisensor data were obtained from the combination of several on-site investigation methods, which were predominantly remote and nondestructive.

### 3.1. Remote Sensing Data Acquisition

Remote sensing methods rely on the satellite or airborne-based sensors, utilized to acquire information on the object or area being investigated.

#### 3.1.1. Unmanned Aerial Vehicle (UAV) for Terrain Topography

To obtain the data on the topography of an investigated terrain in a rapid and efficient manner, an unmanned aerial vehicle (UAV) was employed in this study. UAV provides a privileged aerial point of view that cannot be obtained using terrestrial recordings, which is especially useful along the linear infrastructure, such as flood protection system networks [44], as well in flood management in general [45,46]. In addition, the application of UAV and photogrammetry technology to deliver a high-resolution 3D point cloud is of great benefit for engineering purposes. By taking a predefined number of photographs of the area, which are overlapped in longitudinal and transverse direction, a point cloud is derived.

**Figure 5:**
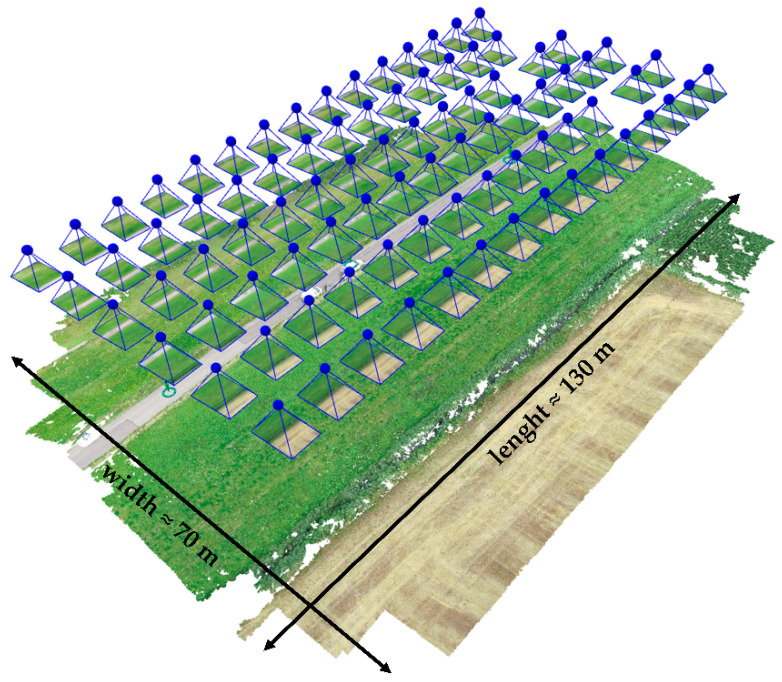
A set-up for UAV photograph acquisition, i.e., defined flying route.

From here, relevant cross-sections of a terrain or an asset can be easily extracted, which enhances and accelerates the overall procedure of defining relevant sections for the numerical analysis. The investigation procedure included preparation of the autonomous flight mission, Figure 5, by defining the area of interest, as well the parameters for flight, which included height, angle of the camera view, longitudinal/side overlapping of the images, and velocity, which were all important for determining the ground sample distance (GSD) of the future model, as the distance between two consecutive pixel centers measured on the ground. Higher GSD values imply lower spatial resolution of the image. When discussing accuracy in aerial mapping, relative and absolute accuracy should be distinguished. While the relative accuracy is linked by comparing features within a reconstruction, the absolute accuracy is related to the true position of a reconstruction in a coordinate system. Since this study overlapped the UAV data with the other spatial data, that is remote sensing measurements, the absolute accuracy was of high importance. Usually, the relative accuracy is considered acceptable if it is within one to three times the ground sampling distance (GSD), while absolute accuracy should be within one to two times the ground sampling distance (GSD) horizontally and one to three times the ground sampling distance (GSD) vertically.

#### 3.1.2. Satellite Monitoring of Ground Displacements

Radar measurements linked to ground-based stations [47,48] or satellites [49,50,51], have been used in recent times to monitor displacements. The latter technology uses satellites that continuously orbit the Earth, where radar sends a pulse down to Earth a few thousand times per second, leading to their reflection from the Earth’s surface and their collection with the satellite. As Mihalinec et al. [52] note, the large benefit of these technologies is the possibility of utilizing them in all weather conditions. Several variants of the radar technologies can be used: (i) synthetic aperture radar (SAR), (ii) InSAR (interferometric synthetic aperture radar), and (iii) DinSAR (differential interferometric synthetic aperture radar). The main difference among these is in the number of used antennas. SAR utilizes one antenna to emit and receive the signal to create high resolution images, while InSAR uses two antennas positioned on the same satellite, where one antenna emits the signal and both antennas receive the return signal, therefore being more accurate than SAR technology. DinSAR technology is more accurate than the previous two variants.

### 3.2. Geophysical Near-Surface Nondestructive Methods

Geophysical methods in general include a series of nondestructive methods to determine the geological–structural and physical–mechanical characteristics of the investigated medium [53]. These methods offer a considerable cost-to-ratio advantage compared to traditional methods, mainly because these investigations provide data of greater volume of the investigated medium, along with relatively cheap instrumentation and speed of the investigation on-site. However, it is important to note that the change in the physical characteristics of the investigated medium must exist so that a particular geophysical method can be considered as acceptable [54].

#### 3.2.1. MASW for Determination of a Small-Strain Soil Stiffness

**Figure 6:**
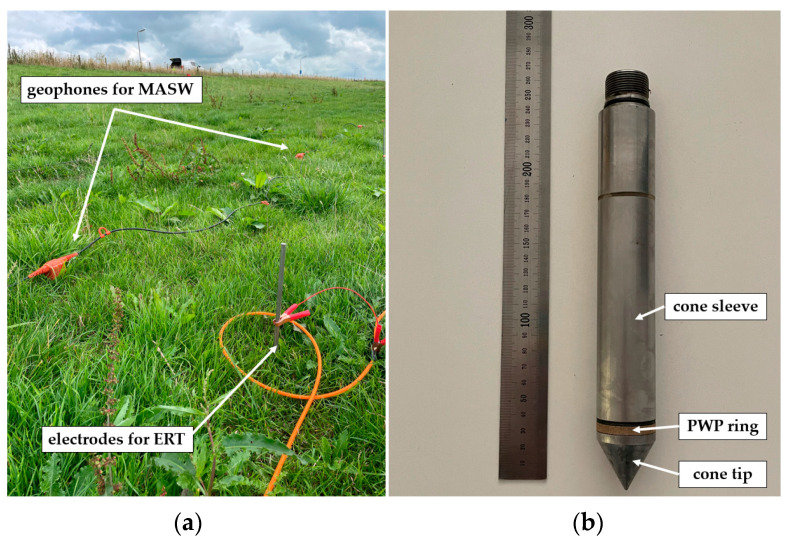
In situ investigation methods: (**a**) MASW geophones and ERT electrodes during the investigation; (**b**) CPT cone parts.

Multichannel analysis of surface waves (MASW) is a nondestructive geophysical method for measuring the velocity of seismic shear waves. It utilizes dispersive characteristics of Rayleigh’s R waves, which propagate to different depths when having different wavelengths or frequencies [55]. As such, MASW is exceptionally useful in determining the elastic modulus of soils at very small strains with respect to depth. During the investigation, several geophones were placed along the investigation line at predefined intervals, Figure 6a. After generating an artificial vertical mechanical impulse on the terrain surface, geophones measured the arrival time of the wave propagating through the soil. During the interpretation phase, a signal collected by the geophones was transformed from the time into the frequency domain [56], and this enabled the determination of the shear velocities along the depth, as well the small strain shear modulus of soil, by utilizing Equation (1).

#### 3.2.2. Electrical Resistivity Tomography (ERT) for Determination of Deposit Thicknesses

Electrical tomography (ERT) provides a more exact picture of electrical resistivity of soil, by registering changes in electrical resistivity in the vertical and horizontal directions. When using the method, the soft clayey soils be identified by very low resistivity, even more pronounced if they are saturated. Thus, the overall thickness of the soft soils, which has high influence on the overall creep behavior, can be identified. The investigation procedure includes positioning of the steel bars, Figure 6a, called electrodes, while the electricity is released into the ground in a non-destructive manner. During the interpretation phase, a 2D electrical tomography profile of apparent resistivities, so-called pseudosections [57], was developed.

#### 3.2.3. Cone Penetration Testing (CPT) for Soil Classification and Determination of Its Physical-Mechanical Parameters

As an alternative to the traditional soil drilling, sampling, and laboratory testing, cone penetration testing (CPT) provides continuous and reliable information along the investigation depth. The CPT is being more and more incorporated into the portfolio of geotechnical engineers due to its major advances in speed of use, as well its repeatability and reliability. This especially comes to the fore when investigating the soil below linear infrastructure such as the embankment and riverbank network [58]. The method relies on pushing a specially designed probe into the soil, Figure 6b, at a relatively fast rate (20 mm/s), thus enabling continuous recording of the cone tip resistance (q_c_) and sleeve resistance (f_s_), as well the groundwater pore pressure (u). Despite the standardized external geometry of the cone, the measurement and transmission system used can vary considerably from one device to another [59]. By utilizing raw CPT collected data, soil layers can be identified using one of the classification procedures, such as [60], as well to provide estimates of its in situ physical and mechanical properties, such as undrained strength, compression modulus, friction angle, and unit weight. The latter correlation, developed by Kovacevic et al. [36], was particularly useful in the present study for determining soil density and for the calculation of small strain stiffness in combination with MASW results, as given in Equation (1).

## 4. Validation of the Methodology—Oostmolendijk Embankment

### 4.1. Description of the Case Study Area

**Figure 7:**
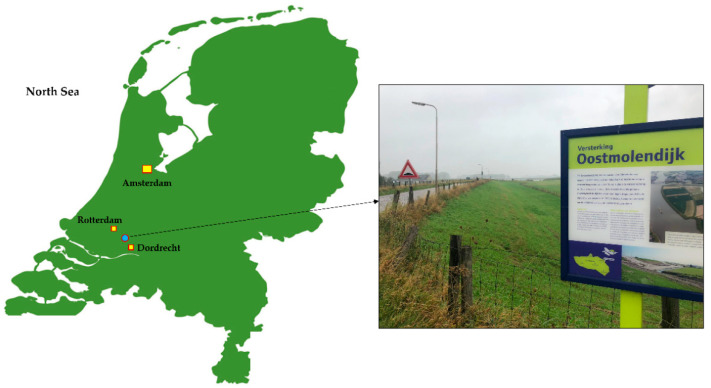
Location of the case study embankment Oostmolendijk.

To validate the proposed methodology based on the implementation of multisensor data in machine learning process, an Oud-IJsselmonde–Oostendam flood protection section in Netherlands was chosen, between Rotterdam and Dordrecht. This embankment section, 9.4 km long and consisting entirely of engineered soil slopes, protects the eastern part of IJsselmonde against the influence of the North River, Figure 7. Particularly, a section named Oostmolendijk was selected for the analysis as it was deemed to be critical due to continuous long-term settlements of the levee crest and slopes.

**Figure 8:**
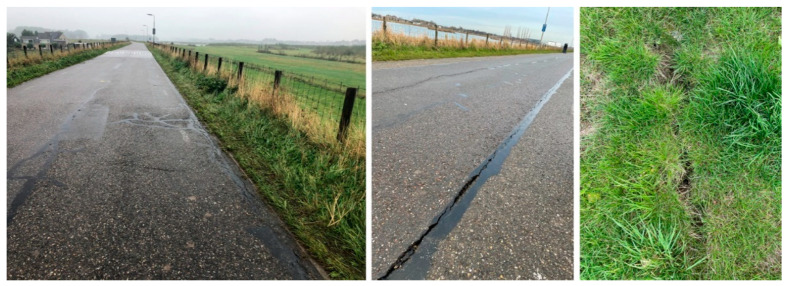
Presence of cracks on the embankment road and slopes.

The time-dependent settlement of the Dutch embankments is a well-known problem, where Speijker et al. [61] note that these embankments slowly sink “away into the sea” due to a combination of factors, such as soil settlement and relative sea level rise. To develop appropriate embankment heightening protocols, the authors proposed the maintenance decision model, validated on the problem of heightening of the Oostmolendijk. Jorissen and Noortwijk [62] highlight the extreme settlement and subsoil consolidation of Oostmolendijk: about 0.60 m in the period from 1969 (first elevation work) to 1981, and about 0.15 m in the period from 1981 to 1989, with only these data available at the time. Second elevation work was conducted in 1991 when the dike was elevated by 30 cm. However, after the 2013 reconstruction work, which included installation of the additional material on the crest and downstream slope, the excessive settlement continued, leading to the present state with many cracks along the road resting on the crest, as well on the slopes, Figure 8. The fact that the embankment body is still in motion and is not stable, raises doubts on the appropriateness of remediation work to increase the crest level, since additional material fill will certainly yield additional loads on the soil.

Speijker et al. [61] estimated the trend of crest level decline by using both a linear and nonlinear approach, for the purpose of calculation of expected costs of Oostmolendijk heightening. Within this study, authors argued that although the assumption of expected crest-level decline being linear in time is quite reasonable when data are lacking, the engineering knowledge suggests the expected crest-level decline to be a logarithmic function of time. Thus, while specifying a nonlinear decline of the embankment and crest, settlement and subsoil consolidations were considered, however without considering effects of creep. Further, the authors reported that for the period of 50 years, a crest height decrease of approximately 1.30 m is expected of which 1.00 m is due to the embankment settlement, and 0.30 m is due to the relative sea level rise.

### 4.2. Conducted Investigations and Obtained Results

**Figure 9:**
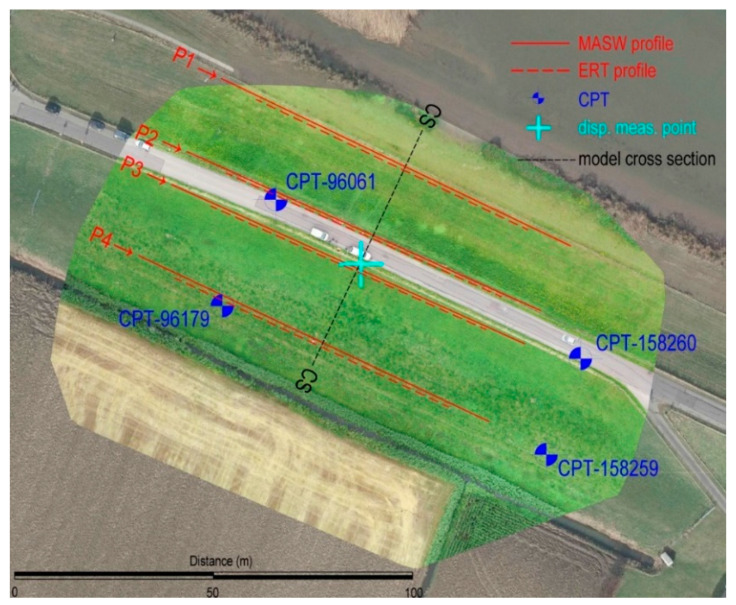
Layout of geotechnical and geophysical investigation work on Oostmolendijk.

To obtain data necessary for the implementation of the methodology, several in situ investigation methods were supplemented by the CPT data from the open-source database DINOloket [63]. The layout of geotechnical and geophysical investigation work is shown in Figure 9, while the obtained data, along with lengths, depths, locations, etc., are shown in Table 1.

**Figure 10:**
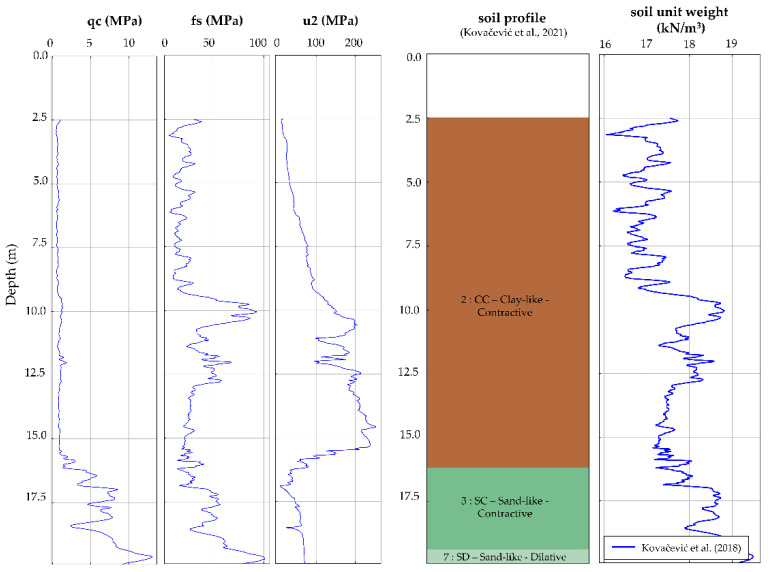
One of Oostmolendijk CPTs with raw qc, fs, and u2 data obtained from CPT_96016 [63], followed by the interpreted soil profile [58] and calculated values of soil unit weight [36].

After obtaining the UAV data, a complete 3D point cloud was developed. In addition to the data given in Table 1, it should be noted that the UAV built-in camera has a sensor width of 13.2 mm and a focal length of 8.8 mm with 5472-pixel image width, all leading to the GSD of 0.83 cm for the flight height of 30 m and camera pointing down to a −90° (with 0° being the horizontal position of a camera). On the ground, three ground control points (GCP) were used for placing model to a local coordinate grid (Amersfoort/RD new, EPSG:28992). Mean absolute error of the investigation was 6 × 10^−6^ m and 1.1 × 10^−4^ m in horizontal (X and Y directions, respectively) and 1.2 × 10^−4^ m vertically (Z direction), providing satisfactory absolute accuracy of the reconstructed model. The development of a point cloud enabled extraction of the relevant cross-section, marked CS in Figure 9, used in the subsequent numerical analysis. Two CPTs were conducted from the Oostmolendijk crest level, while two were conducted on the downstream side. For every CPT investigation, data were continuously recorded with a vertical resolution of 2 cm. This CPT raw data, obtained from the DINOloket [63], were mostly consistent as they point to the clayey material depths up to 15–16 m from the crest level overlying the sandy layers to the investigation depths. The clayey layers showed extremely low values of q_c_ in the clayey layers, up to 1 MPa, while for the sand deposits q_c_ values were in a range from 10 to 20 MPa, Figure 10. By using the Robertson [60] classification and soil layering algorithm developed by Kovačević et al. [58], a soil profile was developed.

**Figure 11:**
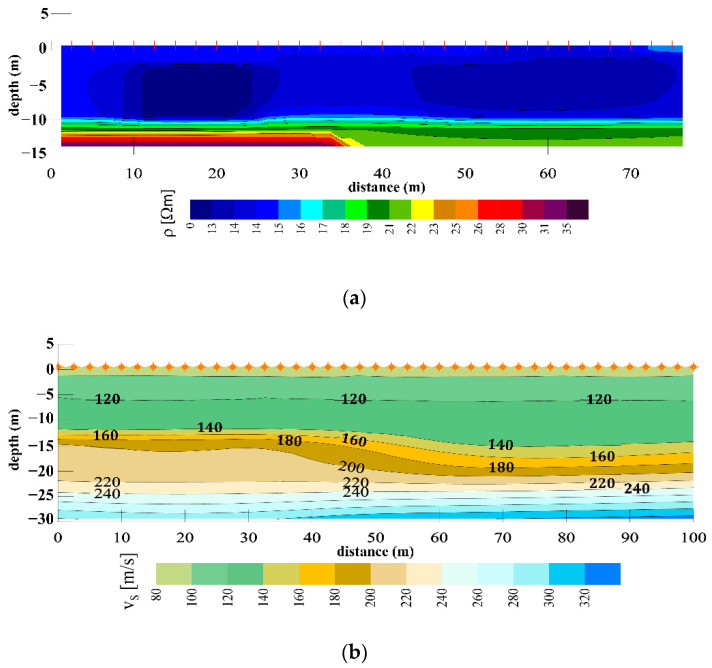
Results of geophysical investigations on profile P1: (**a**) ERT and (**b**) MASW.

Geophysical investigations were conducted on four profiles in a longitudinal direction, as indicated in Figure 9. Each MASW profile consisted of 40 geophones on 2.5 m separation, whereas the each ERT profile utilized 32 electrodes on 2.5 m separation. The ERT investigations showed that the subsoil is formed mostly of the clayey materials with very low resistivities up to 12 to 15 m, below which there is an increase in soil resistivities, attributed to the presence of sand formations, see Figure 11a for profile P1. The ERT profiles on the downstream side also picked up the presence of sandy layers down to a depth of 2 m, which corresponds to the additional material filled during the 2013 rehabilitation work. The MASW results for same profile P1, Figure 11b, show very low v_s_ values (clay) to the depths of 10–15 m below which there is a v_s_ increase (sands).

**Figure 12:**
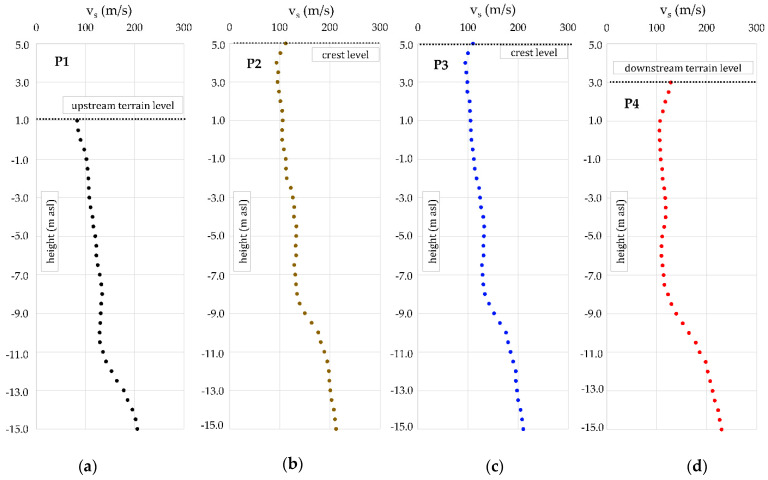
Values of 1D shear velocities from 4 investigation profiles: (**a**) P1 profile on the upstream side; (**b**) P2 on the crest; (**c**) P3 on the crest; (**d**) P4 on the downstream side.

It was also interesting to observe MASW measurements at four single points, along the selected cross-section, marked CS. On all four 1D profiles, Figure 12, a slight decrease of shear velocities can be observed at depths from 7 to 11 m below the embankment toe, which can be eventually attributed to the presence of even softer peat layers within the clay layers.

### 4.3. Long-Term Monitoring Data

**Figure 13:**
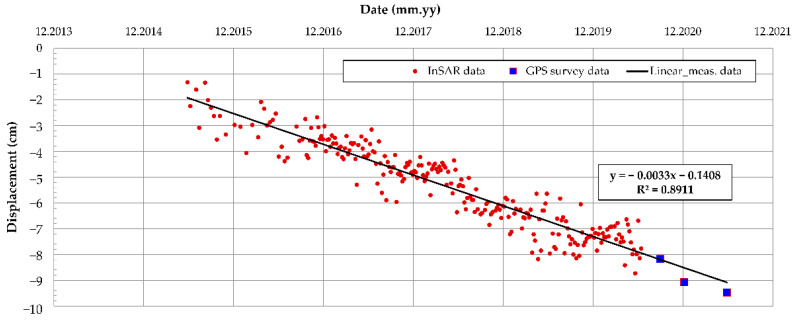
Oostmolendijk displacements in time obtained from InSAR [64] and GPS.

The InSAR data on long-term monitoring of the Oostmolendijk was obtained from the open-access database [64]. By performing mathematical operations on the pulses emitted from the satellite radar and received after their reflection on the Earth’s surface, an image of the settlements containing ten thousand measurements per square kilometer was obtained, covering the whole of the Netherlands. As indicated in the database, the satellite radar looks at the Netherlands from six different positions in space, and the corresponding map layers in the database are called West, Middle, and East; Oostmolendijk monitoring belongs to the West layer. As shown in Table 1, the open database contains measurements from May 2015 to June 2020 and is shown as red points in Figure 13. For this measurement point, displacement data were obtained every 6 days, therefore the point database consisted of 251 measurements. Since reconstruction work by filling additional material ended in the 2013, this marked the starting reference displacement point for the creep analysis. The InSAR measurements from the database [64] usually have a precision of the order of a millimeter. This could, however, vary depending on the location and on location-related noise. The observed point is located on the crest of the Oostmolendijk where the asphalt layer is located, meaning that there is a small influence of the point position to the measurement precision. Regardless, the measurements are of sufficient precision in order to be used in the methodology.

In addition to InSAR data, the additional terrestrial GPS measurement data (blue in Figure 13), were provided courtesy of Waterschap Hollandse Delta, as the infrastructure manager responsible for Oostmolendijk. Even though only three measurements were performed, the obtained data present a continuation of the InSAR database and also point to a clear and continuous ongoing settlement of the embankment. If linear regression is used on a complete dataset, including both InSAR and GPS data, the resulting R^2^ is 0.8911, while the RMSE has value of 0.5628.

### 4.4. Results and Discussion

Once the in situ data were collected and having the displacement measurement database on disposal, the implementation of the research methodology on the Oostmolendijk followed.

#### 4.4.1. Implementation of NetCREEP and PSO

To form a database for the development of a NetCREEP network, many numerical simulations were conducted. Precisely, 125 simulations were carried out with the range (sets) of input parameters. Therefore, the selected ranges were following: (i) for reduction radio r_d_ = [0.10; 0.15; 0.20; 0.25; 0.30]; (ii) Kelvin’s viscosity, η_K_ = [10,000; 20,000; 40,000; 60,000; 80,000]; and (iii) Maxwell’s viscosity, η_M_ = [200,000; 250,000; 300,000; 350,000; 400,000]. These 125 sets (i.e., 5^3^ being the possible number of combinations, where ‘5′ represents a number of predefined values for each creep parameter and ‘3′ represents the number of creep parameters) of input parameters, when used in numerical simulations, resulted in 125 displacements of the crest displacement. The displacement of a crest is marked as relevant for the implementation of methodology since it is exactly at this location where the Oostmolendijk displacements were monitored by InSAR and GPS.

**Figure 14:**
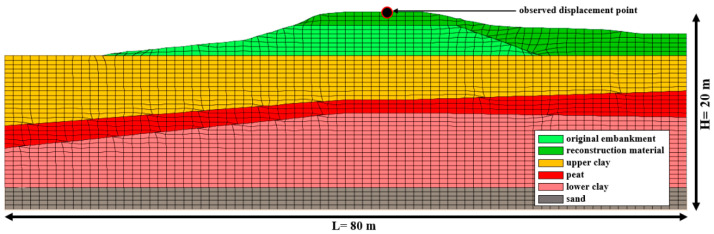
Oostmolendijk numerical model with position of observed displacement point.

Based on the data obtained from investigation works—UAV for terrain topography, shear velocities from MASW and unit weight/density from CPT for small-strain stiffness values, CPT for soil classification and strength parameters, and ERT for thickness of soft clay layers—a numerical model was developed, Figure 14. A sensitivity analysis was performed to check for boundary effects, and a finite difference numerical model, developed in FLAC [35], being 80 m wide and 20 m high was found to satisfy the requirements. Despite the fact that the model divided soft clays into two layers, by introducing a peat layer in between, it should be noted that the same parameters were assigned for upper clay, peat, and lower clay, meaning that the same set of predefined creep parameters were introduced to each layer. Therefore, the division of soft clay in several layers was merely visual, while the assigned parameters were the same. This can be justified by the fact that the peat and soft clays demonstrated similar mechanical behavior. Variations of the q_c_, shown in Figure 10, were relatively insignificant down to the depth of sand layers. When Robertson’s 2016 [60] soil classification was used, which classifies soils based on its behavior, the entire layer was classified as ‘clay-like contractive material’. The case was similar when Robertson’s 2009 [65] soil classification was used, which relies on the textural-based descriptions and where the entire profile up to sand is classified as ‘clay’. It was only when the Dutch modification of Robertson’s 2009 classification was implemented, that some thin layers were classified as peat. This is due to fact that a soil behavior type index (I_c_) in the Dutch modification, with a value of 3.6, is slightly shifted toward higher Q_t_ values in the Q_t_–F_r_ chart. Therefore, it can be stated with great certainty that the mechanical behavior of the soft clay and peat is the same and that these can be evaluated as a single layer in this study. Also, the v_s_ values from Figure 12 show an insignificant decrease in values at depths of 5 to 11 m, yielding the small influence on obtained small strain shear modulus values.

**Figure 15:**
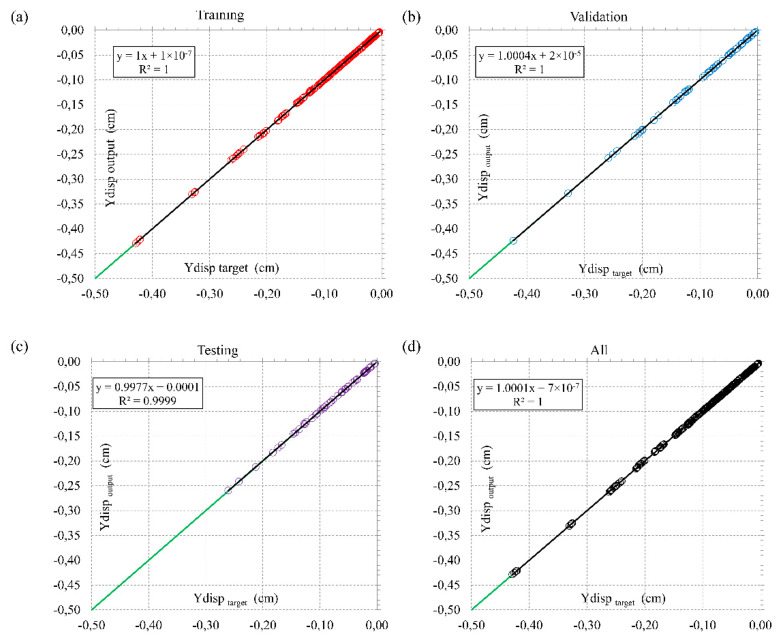
Training (**a**), validation (**b**), testing (**c**), and overall (**d**) datasets with correlation of NetCREEP predicted time-dependent crest displacements and creep parameters.

The input–output pair, provided by the one numerical simulation, is called one dataset. Therefore, by running 125 simulations, 125 input–output pairs were utilized for NetCREEP development, whereas the input was considered as one (r_d_, η_K_, and η_M_) set, and output was considered as a displacement of the crest in 31 observed time (every 3 months from December 2013 to June 2021.). The total number of crest displacements calculated is 3875 (31 observation periods × 125 calculations). The overall matrix output has the form [y^crest^_1_, y^crest^_2_, …, y^crest^_31_]_1_; [y^crest^_1_, y^crest^_2_, …, y^crest^_31_]_2_; …; [y^crest^_1_, y^crest^_2_, …, y^crest^_31_]_125_. Figure 15 shows that R^2^ values for the target-output evaluations for training, validation, testing, and overall datasets are equal to unity. This confirms that the developed NetCREEP established strong correlation between time-dependent crest displacements and creep parameters and that the chosen number of input combinations is representative.

**Figure 16:**
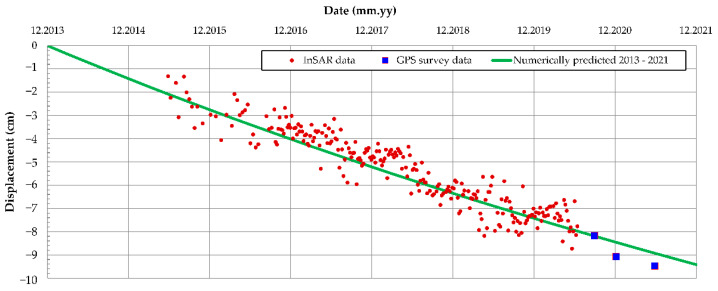
Numerically obtained displacements of the Oostmolendijk for the identified creep parameters.

By having the NN with the architecture adapted to specific problem and by using the particle swarm optimization (PSO) algorithm code, implemented in MATLAB [42], monitoring data from Figure 13 were used to estimate most probable (r_d_, η_K_, and η_M_) set, which would yield observed measurements. The PSO was used for minimizing the sum square error between the network output and the desired output obtained by InSAR and GPS displacement measurements. In the case of Oostmolendijk, the minimum of estimation function (f_min_) equaled to 1.6915 × 10^−6^, whereas the identified values of Burger’s unknown parameters were: (i) r_d_ = 0.2112 (meaning that the large-strain stiffness is 21% of obtained small-strain stiffness), (ii) η_K_ = 48,050, and (iii) η_M_ = 320,000. When these values were implemented in the numerical model, Figure 16, results show that the numerically obtained crest displacement trend correlates well with the crest monitoring results for the period up to June 2021, and this validates the overall methodology.

#### 4.4.2. Prediction of Oostmolendijk Long-Term Behavior

Once the methodology is validated and the most probable creep parameters are identified, a numerical model can be used for prediction of the Oostmolendijk behavior for any required period. Here, the required observed period extends up to 2030, Figure 17. It should be noted that each simulation requires significant long-term computational efforts. While the calculated settlement in June 2021, when the last in situ measurements were conducted, is 8.9 cm, the model predicts the increase of settlement to a value of 16.5 cm by 2030, which is an increase of 85%.

When compared to the different regression functions, used only on monitoring (InSAR and GPS) data, the difference between these functions and the numerically predicted function increases with the increase of observed time. These simple forecasting functions are usually applied in the data analysis to predict a value based on existing values along a predefined trend. This “extrapolation” beyond the “scope of the measured data” is merely to demonstrate what could be expected in terms of time-dependent displacement if only measured data are analyzed. All utilized regression functions, namely linear, logarithmic, and polynomial, have the large R^2^ meaning that there is a clear trend of measured displacements in time. However, once they are extrapolated to 2030, the regression functions overestimate the numerically predicted displacement by 20% (logarithm function), 23% (linear function), or 34% (polynomial function), respectively. Apart from providing more reliable displacement prediction, having the fully defined numerical model means having the fully defined soil stress state for any desired period, something which cannot be obtained by simple extrapolation of the regression functions. Therefore, at some point a soil failure will occur due to excessive deformations and this, unlike with the fully defined numerical model, cannot be predicted by simply extrapolating the measurement data regression functions.

When comparing the numerically obtained results with those from Speijker et al. [61], who estimated the Oostmolendijk long-term crest decline using simplified models with several assumptions, some differences can be observed. By using the linear assumption, Speijker et al. [61] estimated 2.6 cm per year in Oostmolendijk’s crest decline. Here, authors highlight that the expected crest decline in a period of 50 years is 1.30 m, out of which 0.30 m is due to relative sea level rise, and 1.00 m is due to settlement and subsoil consolidation. Since the latter is of interest for this study, it could be concluded that the settlements will occur at a rate of 2 cm per year. When put in context of this study’s analysis, where 2013 is highlighted as the starting observation period after the reconstruction work, by the year 2030, Oostmolendijk would settle up to 34 cm, which is more than twice the value estimated by this study.

The continuous displacement monitoring is of upmost importance for the presented methodology of inverse creep parameter identification. By having a larger monitoring database at our disposal, a more reliable estimation of creep parameters could be obtained, since these are calibrated against the monitoring data. It should be therefore stressed that the continuous monitoring of an embankment not only provides information on its current state and behavior, but also provides a reliable estimate of its future performance.

## 5. Conclusions

The paper presents the methodology for inverse creep parameter identification necessary for a reliable representation of Burger’s constitutive law. In order to predict the long-term creep behavior of soft soils, the methodology consists of several phases, with the overall aim to develop a neural network optimized by the particle swarm algorithm, so that most probable creep parameters are identified, overcoming the issues of costly and time-consuming laboratory creep tests. A NetCREEP network is trained, validated, and tested on the large number of input–output sets obtained from the numerical analyses, whereas the input for the development of a numerical model was obtained by multisensor data, namely UAV for terrain topography, shear velocities from MASW and unit weight/density from CPT for small-strain stiffness values, CPT for soil classification and strength parameters, and ERT for thickness of soft clay layers. Once the numerical model was defined, the predefined sets of Burger’s unknown parameters, stiffness reduction ratio (r_d_), Kelvin’s viscosity (η_K_), and Maxwell’s viscosity (η_M_) were assigned to the model. The obtained numerical input–output datasets provided the basis for the development of NetCREEP, which, after several iterations, consisted of ten nodes distributed within the total of four hidden layers. By utilizing the PSO, the most probable set of creep parameters was identified where the methodology took full advantage of the database consisting of the continuous monitoring of long-term soil displacements, obtained by InSAR and GPS. The overall methodology was verified on a case study location of an Oostmolendijk embankment in the Netherlands, well-known for its extreme settlements. The developed and PSO-optimized NetCREEP provided a set of creep parameters, which enabled the numerical simulation of a monitoring database, as well the prediction of the long-term behavior of an embankment. Within this procedure, PSO yielded sufficiently low values of minimum of estimation function. It was shown that the prediction model provides 20–35% lower values of long-term settlements when compared to traditional statistical regression functions extrapolated to the desired period. When compared to some previous studies on the embankment settlements, this study yields twice the lower values. A fully defined numerical model, with a best estimate of input creep parameters, also provides the insight into long-term soil stress state, which cannot be obtained by simple extrapolation of the regression functions used merely on monitoring data. The study also demonstrates the huge benefits of having the continuous monitoring of the embankment, not just for the current assessment of its behavior, but also for reliable prediction of future performance. For this purpose, remote sensing InSAR data can be extremely beneficial.

## Figures and Tables

**Figure 17:**
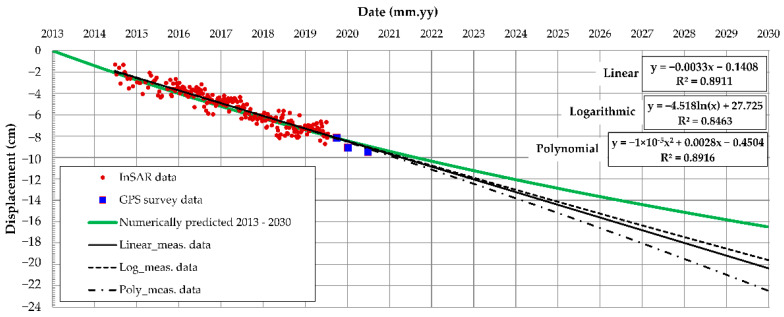
Numerically predicted displacements of Oostmolendijk by the year 2030.

**Table 1:** Overview of the Oostmolendijk investigation methods and displacement monitoring.

**Soil Layering/Parameters**							
Type	Total no. of investigation profiles or points	No. of profiles (points) on the crest	No. of profiles (points) upstream/downstream		Length of each profile (m)	Depth of investigation (m)	Source
MASW	4	2	1/1		100	26	In situ
ERT	4	2	1/1		75	15	In situ
CPT	4	2	0/2		-	20	[63]
**Terrain Topography**							
Type	Flight height (m)	Scanned area (m × m)	Photo overlapping	No. of photos	No. of 3D points (million)	GSD (cm)	Source
UAV	30	70 × 130	front 70% side 70%	87	63.6	0.83	In situ
**Displacement Measurement**							
Type	Measurement period	Satellite	Point ID from database [56]		Coordinates (EPSG:28992) of measurement point *	No. of measurements	Source
InSAR	from May 2015 to June 2020	WEST-1	L00019660P00016545		N 102968.0 E 430885.0	251	[64]
GPS	from June 2020 to June 2021	-	-		N 102969.6 E 430885.8	3	IM **

* GPS monitoring point was chosen within the GPS database as the one closest to the InSAR monitoring point (4.1 m distance between GPS and InSAR monitoring point). ** provided by the courtesy of Infrastructure Manager Waterschap Hollandse Delta.
